# Diffuse primary extra-gastrointestinal stromal tumor of the peritoneum: A rare case report

**DOI:** 10.1097/MD.0000000000022493

**Published:** 2020-10-02

**Authors:** Liang-ji Lu, Xiao-pei Xu, Fei Dong, Jie Min

**Affiliations:** a Department of Radiology, the Second Affiliated Hospital, Zhejiang University School of Medicine; b Department of Radiology, the Second Affiliated Hospital, Zhejiang University School of Medicine, No. 88 Jiefang Road, Hangzhou, Zhejiang Province, China.

**Keywords:** extra-gastrointestinal stromal tumors, imatinib mesylate, peritoneum

## Abstract

**Rationale:**

Gastrointestinal stromal tumors that present outside the gastrointestinal tract are known for extra-gastrointestinal stromal tumors (EGISTs) and they share the same morphological and immunohistochemical characteristics with gastrointestinal stromal tumors. Here we report a rare case of diffuse primary EGIST arising at peritoneum.

**Patient concerns:**

A 57-year-old male presented to the hospital with abdominal pain and right lower abdominal tenderness.

**Diagnosis:**

The core needle puncture biopsy showed epithelial-like cells and the nuclei were ovoid and focally elongated. Immunohistochemical examination was consistent with a primary EGIST of the peritoneum.

**Interventions:**

The patient was treated with Imatinib mesylate.

**Outcomes:**

Five months later, there is no complication resulting from treatment. The follow-up abdominal contrast-enhanced CT showed the lesion was significantly decreased in size, and was evaluated as partial response. The patient continued the treatment with Imatinib as prescribed by the oncologist.

**Lessons:**

EGISTs are rare and should be considered in the differential diagnosis of the peritoneal tumors and immunohistochemistry helps to confirm the diagnosis. Further study with longer follow-up is desired to characterize these uncommon tumors.

## 1 Introduction

Gastrointestinal stromal tumors (GISTs) represent a distinct group of tumors that originate from Cajal interstitial cells (CIC), which normally express CD117–the tyrosine kinase component of the mass factor receptor in the stem cell.^[1]^ GISTs can appear anywhere in the gastrointestinal tract but usually involve the stomach with a prevalence around 60% or the small intestine in 30% of all cases.[^2^,^3^] Colon, rectum and esophagus are usually less involved and GISTs arising as primary tumors outside the gastrointestinal tract are called extra-gastrointestinal stromal tumor (EGIST) and they compromise less than 5% of all GISTs. The clinical features of GISTs can be heterogeneous, with tumor size and mitotic activity being the most useful characteristics of metastatic risk stratification.^[4]^

The origin of EGISTs is uncertain, but EGISTs usually have identical histological and immunohistochemical characteristics with GIST, and they may occur in mesentery, omentum, and retroperitoneum in very rare cases. So, they are believed to represent either GISTs that were separated from the gastrointestinal tract wall,^[5]^ or independent growths of mesenchymal cells of the omentum and mesentery from which they originate. EGISTs arising in the mesentery and omentum account for approximately 2% and 1% of all GISTs, respectively.^[6]^ Omental EGISTs were reported as solitary or multiple tumors and their aggressiveness ranged from benign to metastatic behavior.

Of all the EGISTs, diffuse primary EGISTs in the peritoneum are rarely reported in past literatures. Arabi et al^[7]^ once presented an unusual case of EGIST that presented with multiple gooseberry-like nodules involving the whole abdominal cavity, the omentum, peritoneum and small bowel mesentery. Thus, our present study reports a rare case of a diffuse primary peritoneal EGIST in abdomen and pelvis.

## 2 Case presentation

### 2.1 Clinical findings

A 57-year-old male presented with vague paroxysmal lower abdominal pain under no obvious predisposing causes. The pain lasted for a week and there was no symptom like nausea, vomiting, fever or ague. No history of bowel habit changes, fevers, or weight loss were found. The patient denied any abdominal trauma or past surgeries and his family history was unremarkable. A physical examination revealed right lower abdominal tenderness and suspicious rebound pain.

Blood tests revealed increased hemoglobin (129 g/L), platelet count (312 × 10^9^/L), monocyte percentage (12.4%), carbohydrate antigen 125 (181.3 U/ml) and C reactive protein (17.5 mg/L).

### 2.2 Imaging findings

**Figure 1:**
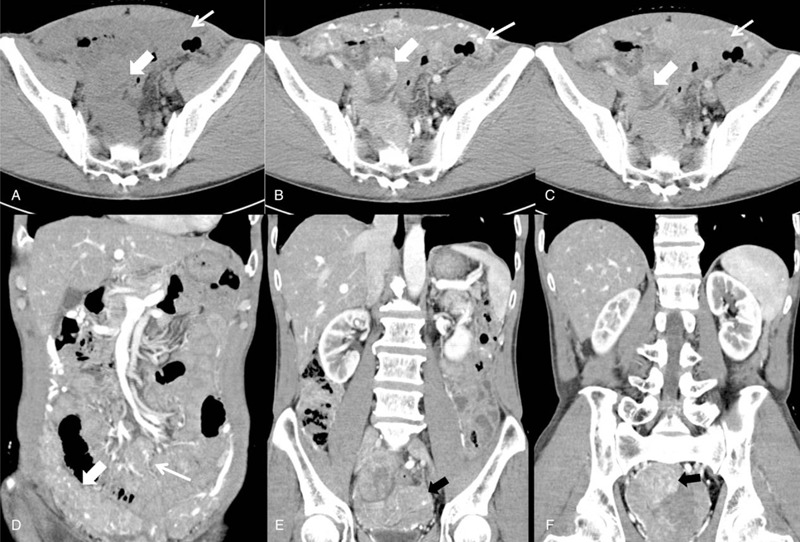
A: Axial view plain CT scan showed a large soft tissue mass involving the pelvic peritoneum (thick arrow) and greater omentum (thin arrow); B: contrast-enhanced CT scan showed significant enhancement during arterial phase (thick arrow) and dilated feeding artery supporting the omental lesion (thin arrow); C: non-enhanced necrotic area (thick arrow) could be seen within the pelvic lesion during parenchymal phase, and the omental lesion demonstrated a homogeneous enhancement; D-F: coronal view of greater omental (thick arrow), mesentery (thin arrow) and pelvic (black arrow) lesions.

Contrast-enhanced computed tomography (CE-CT) was performed for initial examination. CT showed a large soft tissue mass with wide involvement of greater omentum, mesentery, peritoneum of abdomen and pelvis, but spared gastrointestinal tracts and appendix. The component was heterogeneous with high density area and peripheral contrast enhancement. Part of the lesion had nodular appearance and presented fusion of plaques. The lesion had significant enhancement after intra venous contrast administration and dilated feeding arteries could be seen near the lesion (Fig. 1). The CT value of the EGIST was 40HU for plain scan, 83HU for arterial phase and 80 for parenchymal phase. Patchy shadow with no enhancement could be seen within the lesion and indicated the existence of colliquative necrosis area.

**Figure 2:**
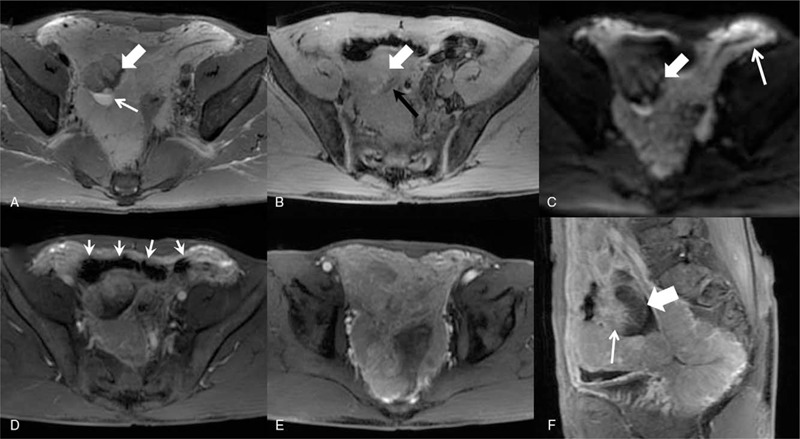
A: axial view of T2WI showed a pelvic hyperintense lesion with hypointense bleeding area (thick arrow) and capsule, as well as focal cystic change (thin arrow); B: axial view of T1WI revealed hyperintense bleeding area (thick arrow) within the pelvic lesion and hypointense capsule (thin arrow) surrounding the lesion; C: axial view of DWI showed pelvic lesion with mixed signal (thick arrow) and greater omental lesion with restricted diffusion (thin arrow); D: axial view of contrast-enhanced MRI showed a clear boundary between the lesion and non-enhance hypointense small intestine (thin arrow); E: significantly enhanced pelvic and greater omental lesion; F: sagittal view revealed the pelvic necrosis area (thick arrow) and enhanced area (thin arrow).

Further contrast-enhanced magnetic resonance imaging (CE-MRI) was arranged for the patients. The lesion presented isointense on T1-WI and hyperintense on T2-WI. Restricted diffusion could be seen on diffusion weighted images. A thread like hypointense capsule could be identified around the lesion on T1-WI and T2-WI (Fig. 2). The lesion had a clear boundary with surrounding tissues. Contrast-enhance images showed significant enhancement and dilated feeding arteries were clearly seen near the lesion. Patchy shadow with no enhancement could be seen within the lesion and indicated the existence of colliquative necrosis area.

### 2.3 Histological findings

After a multidisciplinary team discussion, the nature of the lesion was hard to determine, thus a core needle puncture biopsy was recommended. Ultrasound-guided biopsy of pelvic mass was conducted, and a microscopic examination showed epithelial-like cells. The nuclei were ovoid and focally elongated. The mitotic figures were more than 5 per 50 high power fields (HPF). Vascular space involvement could also be seen in the lesion. Immunohistochemically, the tumor cells were positive for cluster of differentiation (CD) 117, syn, DOG-1, Vimentin, Ki-67 (≥10%) and negative for CD34, S100. The mutation detection showed mutually exclusive gain-of-function KIT platelet-derived growth factor receptor-alpha (PDGFRA) mutations. The described microscopic features, correlated with the macroscopic description, size and relative position to the digestive tract corresponds to a GIST with malignant behavior (high-risk EGIST, 3b prognostic group can be reported based on tumor size and mitotic counts on histology and immunohistochemistry), developed outside the digestive tract, without contact to the digestive lumen. Due to the diffusive nature of current case, resection of the tumor could not be carried out and Imatinib mesylate therapy was conducted afterwards.

**Figure 3:**
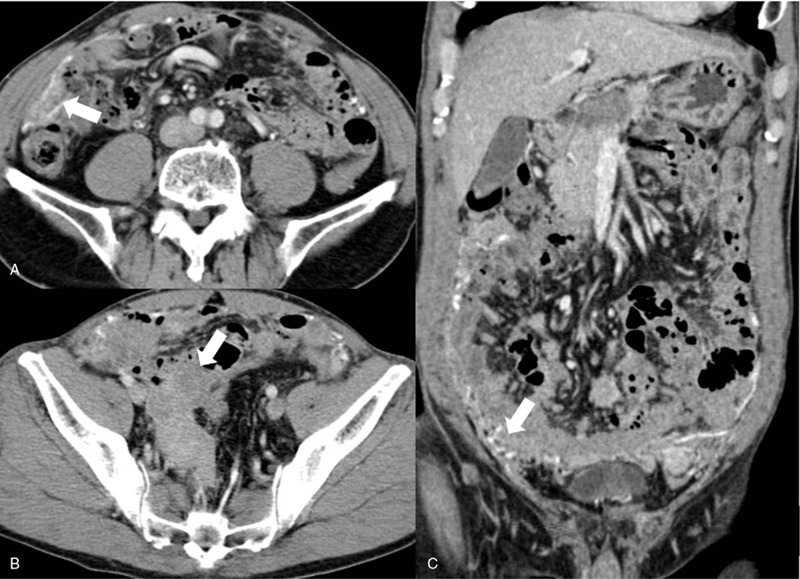
After 5 months treatment of Imatinib, follow-up abdominal CT images (A-C) showed that both pelvic and omental lesions (thick arrow) significantly decreased in size.

The patient was treated with Imatinib (400 mg/qd), and experienced the mild incidence of diarrhea without other adverse effects. Five months later, the follow-up abdominal contrast-enhanced CT showed the lesion was significantly decreased in size, and was evaluated as partial response (Fig. 3). To achieve complete response, the patient continued the treatment with Imatinib as prescribed by the oncologist.

### 2.4 Ethical approval

Written informed consent was obtained from the patient and approval of this case study was obtained from the Institutional Review Board of the second affiliated hospital of Zhejiang university school of medicine.

## 3 Discussion

EGISTs are a group of rare tumors with similar histology and immunohistochemical features to GISTs, occurring outside the gastrointestinal tract, majority of them in the omentum and mesentery or in the retroperitoneum.^[3]^ It is revealed that less than 100 cases of EGISTs had been reported worldwide.[^7^,^8^] Like their digestive counterparts, most EGISTs tumors are typically positive for CD117 and less consistently for CD34, positive for *α*-SMA and negative for desmin and S100 protein.[^9^,^10^,^11^,^12^] The case we reported in current study demonstrated positive immune staining for CD117, syn, DOG-1, Vimentin, and Ki-67, which are in accordance with EGIST diagnosis. But what makes our case unique is the primary site of the tumor. The EGISTs reported by past studies were often localized in greater or smaller omentum. In current study, the EGIST is extensive in nature and had a wide involvement of greater omentum, mesentery, peritoneum of abdomen and pelvis. The extensive nature made part of the lesion demonstrated a fusion appearance and colliquative necrosis area could be seen in the lesion (Fig. 2). This is confirmed by the contrast-enhanced imaging which showed significant enhancement of the lesion and large nutrient artery supporting the lesion.

Although the contribution of imaging techniques (CT scan and MRI) is undeniable, preoperative diagnosis of EGISTs is difficult because the exact primary site of the process is not always easily recognized.^[9]^ When we found a thread like hypointense capsule around the lesion, it usually indicated that the lesion had a clear boundary with surrounding tissues. Contrast-enhance images showed significant enhancement and dilated feeding arteries were clearly seen near the lesion. Patchy shadow with no enhancement could be seen within the lesion and indicated the existence of colliquative necrosis area. With those imaging features, we should always consider GIST. However, EGISTs were often large size due to their anatomic site, having enough space to grow before producing symptoms.[^13^,^14^] In our case, the patient only had vague paroxysmal abdominal pain, discomfort and distention although the tumor was extremely diffused in peritoneum. The grading system defined by a combination of mitotic rate and tumor size, which is commonly used in GIST, may not be applicable in EGIST. Yamamoto et al^[15]^ have defined 3 prognosis categories of EGISTs on the basis of a combination of the mitotic rate and Ki-67 proliferation index tumor: a high-risk group (≥5/50 HPF with ≥10% Ki-67), an intermediate-risk group (≥5/50 HPF with <10% Ki-67 or <5/50 HPF with ≥10%Ki-67), and a low-risk group (<5/50 HPF with <10% Ki-67). Based on this, our case can be defined as high risk presenting >5/50 HPF with 10% Ki-67 expression.

Considering the clinical administration, surgical removal remains the standard treatment for nonmetastatic EGISTs.^[10]^ There is no consensus regarding adjuvant therapy in such cases. Chemotherapy and radiotherapy showed limited benefit.^[16]^ Imatinib (STI-571), a tyrosine kinase inhibitor known to inhibit the activities of KIT and PDGFRA, is currently the treatment of choice for metastatic and unresectable GISTs. More recently, imatinib is also recommended to be used as adjuvant therapy after a complete respective surgery of high-risk GISTs in order to prevent recurrences.^[13]^ Although the treatment was recommended to last for 1 year, in a recent randomized trial, treatment for 3 years showed a more favorable recurrence-free survival than that of the 1- year treatment. In our case, Imatinib mesylate therapy was conducted due to the wide involvement of the tumor.

## 4 Conclusion

EGIST is a rare tumor, and diffuse primary EGISTs of peritoneum is even more rare. When radiologists discover diffuse peritoneal lesion with focal fusion necrosis, EGIST should be considered as one of the primary differential diagnosis. Complete surgical resection is the only effective modality despite significant advances in new chemotherapeutic drugs. However, when the lesion is too diffusive to remove, targeting drugs like Imatinib might be an option. Accumulating data and extended future studies are necessary to better define EGISTs, their pathogenies, behavior and treatment.

## Author contributions

**Methodology:** Xiao-pei Xu.

**Resources:** Fei Dong.

**Software:** Fei Dong.

**Supervision:** Jie Min.

**Writing – original draft:** Liang-ji Lu.

**Writing – review & editing:** Jie Min.
